# Double burden of maternal and child malnutrition and socioeconomic status in urban Sri Lanka

**DOI:** 10.1371/journal.pone.0224222

**Published:** 2019-10-22

**Authors:** Chisa Shinsugi, Deepa Gunasekara, N. K. Gunawardena, Wasanthi Subasinghe, Miki Miyoshi, Satoshi Kaneko, Hidemi Takimoto

**Affiliations:** 1 National Institute of Health and Nutrition, National Institutes of Biomedical Innovation, Health and Nutrition, Tokyo, Japan; 2 Department of Global Health Promotion, Tokyo Medical and Dental University, Tokyo, Japan; 3 Department of Biochemistry and Clinical Chemistry, Faculty of Medicine, University of Kelaniya, Ragama, Sri Lanka; 4 Department of Parasitology, Faculty of Medicine, University of Kelaniya, Ragama, Sri Lanka; 5 Department of Nutrition, Aomori University of Health and Welfare, Aomori, Japan; 6 Department of Eco-epidemiology, Institute of Tropical Medicine, Nagasaki University, Nagasaki, Japan; Universidade de Sao Paulo, BRAZIL

## Abstract

Child malnutrition and maternal obesity are serious public health issues in Sri Lanka. This study explores the associations between socioeconomic status and the double burden of malnutrition among school-aged children and within their household. A total of 543 primary school children aged 5–10 years (204 boys and 339 girls) in Gampaha District, Sri Lanka, were included in the analysis. The nutritional statuses of thinness, normal, overweight, and obesity for children and mothers were defined according to WHO growth references and body mass index. Maternal education, household equivalent income, and maternal employment were used as socioeconomic status indicators. The proportion of child thinness and overweight was 19.3% and 13.4%, respectively, and that of maternal overweight (body mass index ≥ 25 kg/m^2^) was 36.5%. A positive correlation was found between maternal body mass index and the child’s body mass index for age z-score in older boys and younger girls. A multivariate stepwise logistic regression analysis showed that lower education of mothers posed a higher association with child thinness (adjusted odds ratio = 2.33, 95% confidence interval: 1.08–5.00). Mothers with overweight and obesity were less likely to have a child with thinness (adjusted odds ratio = 0.30, 95% confidence interval: 0.16–0.58). Maternal employment status and household equivalent income were not significantly, but marginally, associated with child overweight and obesity. Socioeconomic inequality combined with maternal nutritional status affected child malnutrition. These findings suggest that the underlying circumstances within households should be considered to improve child malnutrition.

## Introduction

Child malnutrition remains a serious public health concern in Sri Lanka [1, 2]. Malnutrition (underweight or overweight/obesity) in school-aged children, not only in children under five years, may continue to pose an increased risk for diseases, cognitive delay, poor academic performance, and lower productivity in later life [3]. In 2015, the Annual Health Bulletin stated that the proportion of wasting (-2 standard deviations (SD) below weight-for-height) and stunting (-2 SD below height-for-age) in the first grade of primary school children (5–6 years old) was 20.3% and 8.7%, respectively [4]. It is essential to prevent delayed growth-related nutritional deficiency in school children to facilitate a better future.

Maternal obesity has been recognized as a global pandemic, especially in urban areas in low- and middle-income countries. With the socio-economic transition in Sri Lanka that followed the end of the long-lasting civil war in 2009, most deaths now occur due to chronic non-communicable diseases (NCDs), including diabetes [4]. The 2016 Sri Lanka Demographic and Health Survey (DHS) showed that 45.3% of married women of reproductive age (15–49 years) are overweight or obese (body mass index (BMI) > 25 kg/m^2^) and, particularly, this percentage has increased among women living in urban areas (55.8%) [5].

The coexistence of under- and over-nutrition is referred to as the double burden of malnutrition, and has been reported worldwide [6, 7]. This phenomenon could also occur at the household level, that is, the co-existence of child undernutrition and maternal overweight or obesity and may be caused by differences in the household environment. Given that socially vulnerable families may not be able to consider the imbalances in nutritional intake and food expenditure within the household, it is useful to determine the bottle-neck effect of specific socioeconomic factors, including maternal social status. To address the rapid increase in adult obesity, it is also important to educate children to maintain their diet healthy.

Socioeconomic status (SES) factors, including maternal education in Mexico [8], and in Asia and other countries [9], community and household economic status in Kenya and in low- and middle-income countries [7, 10], and maternal employment status in the UK [11], have been found to be important predictors of malnutrition in children. However, in Sri Lanka, little is known about the relationship between SES and the double burden of malnutrition in primary school-aged children and their mothers. A study in plantation communities observed the relationship between maternal employment and undernutrition of primary school children [1], but the majority of the residents were employed as unskilled laborers and therefore, the working environment is obviously different from the urban setting. Thus, the present study aimed to investigate the association between SES and the nutritional statuses of mothers and their children in urban Sri Lanka.

## Methods

### Study population

This cross-sectional study on primary school children (Grade 1–5) was conducted in September 2017 in Gampaha District, Sri Lanka. This district is the second most populated district (population 2,354,000) in Sri Lanka and is located in the northeast of Colombo [4]. It has undergone rapid urbanization owing to recent economic growth, including infrastructure improvements.

**Fig 1:**
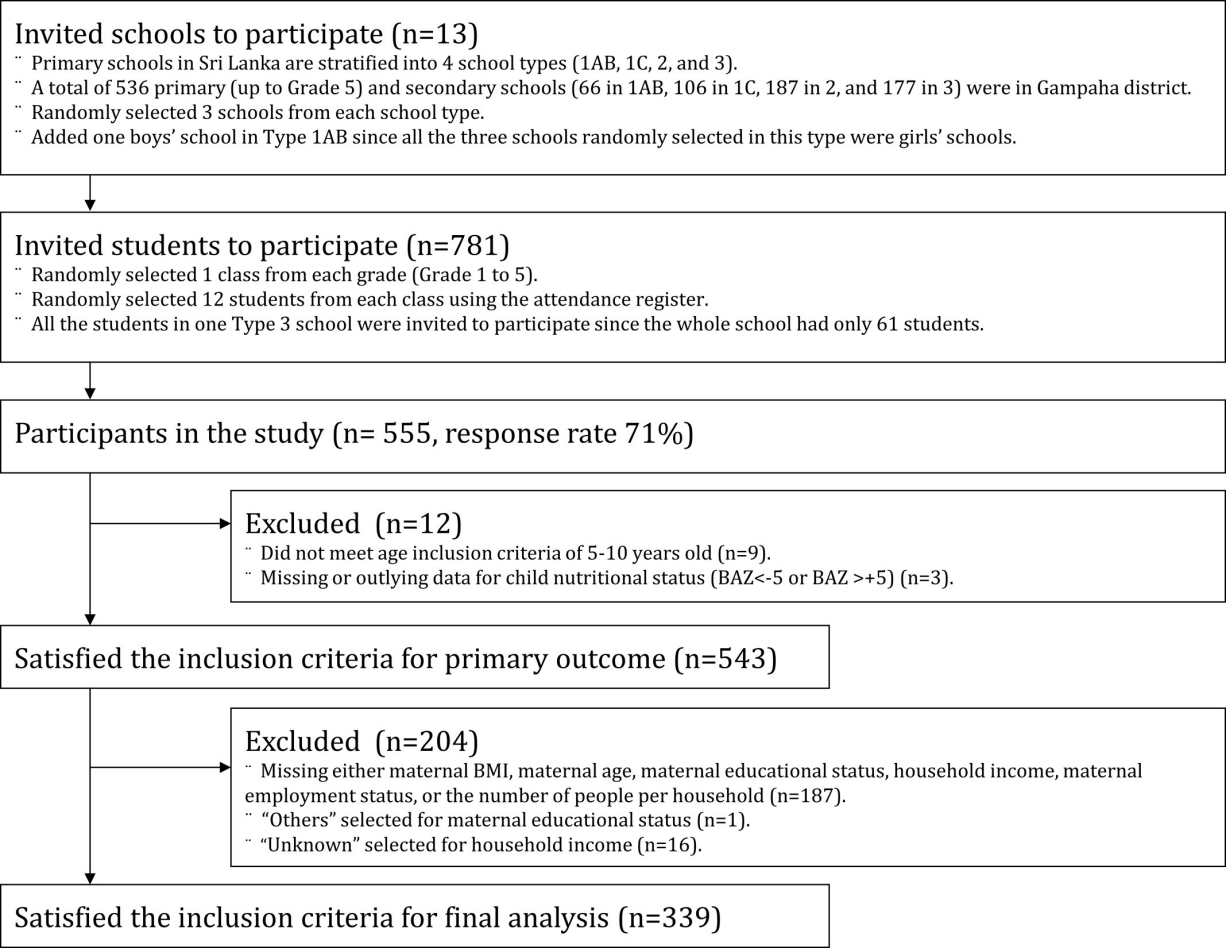
Participant flow chart for the analytic sample.

Stratified random sampling was applied to select study participants. In the educational system of primary (up to Grade 5) and secondary schools in Sri Lanka, schools are stratified into the following four types: Type 1AB (13-year education with arts and science), Type 1C (13-year education with arts), Type 2 (11-year education), and Type 3 (5-year education). From a list of all the primary schools in Gampaha district (n = 536), three schools from each of the four types were selected randomly. One boys’ school was added in Type 1AB as all the three selected schools in this type were girls’ schools. Thus, 13 schools were selected in total. For each grade in the selected schools, one class was randomly selected. For each class in the grade, 12 students were also randomly selected from the index number of the attendance register. These random selections were conducted using a random number table. As one of the 13 schools had only 61 students in total, all the students in that school were included in the study. After securing prior permission from the relevant authorities, informed written consent was obtained from the parents or guardians of the children before data collection. Anthropometric measurements of students were performed at each school premises by a trained team of two staff members lead by a Bachelor of Medicine and Bachelor of Surgery (MBBS) qualified lecturer following a standard operating procedure. Validation of the questionnaire was not conducted since the questions used in this study were created based on the Sri Lanka DHS [5]. Sociodemographic factors were collected using a self-administered questionnaire, which was written in participants’ native language and was distributed among the parents or guardians. Of the 555 students who participated in the study (participation rate: 71%), the analysis was performed on the data collected from 543 children (204 boys, 339 girls), after excluding nine children whose age was not within 5–10 years age group (early admission or failure of upgrade), and three children who had outlying or missing data on the outcome variables (See Fig 1).

### Data collection

#### Anthropometric measurements

Children’s height and weight were measured to the nearest 0.1 cm and 0.1 kg respectively using a stadiometer (SECA China) and a digital scale (SECA China), whilst barefoot and wearing light-weight clothes. BMI-for-age z-score (BAZ) was calculated to determine whether the nutritional status of the child was normal or malnourished (thinness, overweight, or obesity), according to the WHO growth references for children aged 5–19 years old [12]. Thinness was defined where BAZ was less than -2 standard deviations (SD) from the median (BAZ < -2), whereas overweight was defined where BAZ was more than +1 SD from the median (BAZ > +1). Obesity was defined if BAZ was greater than +2 SD above the median (BAZ > +2). Age in months was determined by subtracting the child’s birthdate from the study date, dividing the number of days by 30.4375, and then rounding it to the nearest month. Maternal BMI was categorized as being either underweight (BMI < 18.5 kg/m^2^), normal (BMI 18.5–<25 kg/m^2^), overweight (BMI 25–<30 kg/m^2^), or obese (BMI ≥ 30 kg/m^2^) [5]. BMI (kg/m^2^) was calculated as weight (in kilograms), divided by the square of height (in meters). The weight and height of the students’ mothers were recorded by self-report, or otherwise these were verified / measured along with the child’s using the same scales.

#### SES

Maternal education, household income, and maternal employment status were used as indicators of SES. For maternal education, respondents selected their educational status from the following: no education, incomplete primary, complete primary, incomplete secondary, complete secondary, higher, or others. For household income, participants were asked to select their monthly average income in Sri Lankan Rupees (Rs; USD 1 ≈ Rs 150, as of September 2017) from the following categories: less than Rs 7,500, Rs 7,500–<15,000, Rs 15,000–<Rs 50,000, Rs 50,000 or more, or unknown. For maternal employment status, the choices were housewife, government, private sector, self-employment, agriculture, schooling, job-hunting, unemployment or other.

#### Statistical analysis

For the further analyses to examine the relationship between maternal and child nutritional status and SES, data on 339 children were obtained after excluding: missing values of either maternal BMI, maternal age, maternal educational status, household income, maternal employment status, and the number of people per household; where maternal educational status was recorded as others; or where household income was recorded as unknown (Fig 1). Trends in children’s nutritional status across maternal BMI categories were assessed.

SES was re-categorized as described in the following sentences. Maternal educational status was divided into the following three categories: low (no education, incomplete primary, or complete primary), middle (incomplete secondary), or high (complete secondary or higher). Household equivalent income (HEI) was calculated as monthly household income divided by the square root of the number of people per household and sorted into the following three categories: low (less than Rs 15,000), middle (Rs 15,000–< Rs 50,000), or high (Rs 50,000 or more). Maternal employment status was divided into the following three categories: housewife, employed (government, private sector, and self-employment), or others (schooling, job-hunting, unemployment and other).

The potential association between demographic characteristics, including SES factors, and nutritional status among children, was investigated using the trend test, stratified by maternal BMI categories (normal or overweight/obesity). Participants with the maternal BMI category “thinness” were omitted because they only accounted for 7.4% (25 mothers) of the sample population. For final analyses, the dependent variables were the nutritional status of child (thinness, normal, and overweight and obese), whereas independent variables were SES indicators (maternal education, HEI, and maternal employment status) and the nutritional status of the mothers (thinness, normal, and overweight and obese). The statistical significance level was set at *p* < 0.05. Crude analysis (Model 1) and multiple logistic regression analyses with backward stepwise selections (Model 2) were conducted to examine the relationship between child’s nutritional status and SES, and maternal nutritional status adjusted for child sex and maternal age. Initially, we stratified both mothers’ and children’s nutritional status, because we speculated that child nutritional status is separately affected according to current maternal nutritional status. However, stratifying by current maternal nutritional status leads to small sample size in each children’s nutritional category. Applying multiple regression models enables to adjust the effects of all the factors entered into the model simultaneously, therefore we included maternal nutritional status as an independent variable, instead of stratifying according to maternal nutritional status. A significance level of 0.35 was applied for removal from the model (Model 2) to control confounding factors. All the selected variables were used for calculating adjusted odds ratios (aOR). The statistical software Stata Macro Package version 15.1 (Stata Corporation, TX, USA) was used for all analyses.

This study was conducted after receiving approval from the Ethics Review Committee of the Faculty of Medicine, University of Kelaniya, Sri Lanka, and the Institutional Review Board of the National Institutes of Biomedical Innovation, Health and Nutrition, Japan (No. 150). Permission for the study was also obtained from the Department of Education, Western Province, Sri Lanka.

## Results

The demographic characteristics of the study participants are presented in Table 1. The mean age of the children ± SD was 7.6 ± 1.5 years. Their mean height and weight were 123.7 ± 9.2 cm and 23.4 ± 6.7 kg, respectively. The percentages of thinness and overweight (including obese) among children were 19.3% and 13.4%, respectively, while the percentages of thinness and overweight (including obese) in mothers were 5.0% and 36.5%, respectively. Ethnically, the majority of the participants were Sinhala. Regarding religion, 68.1% were Buddhist and 28.2% were Christian. Almost half of the mothers were in their 30s, and about 30% were in their 40s. Approximately 70% were housewives, whereas 22.1% were employed outside the home. As to maternal educational status, most (43.1%) had graduated from secondary school or had received higher education. A total of 23.6% had a monthly household income of less than Rs 15,000; 58.2% had an income between Rs 15,000 and Rs 49,999; and 11.2% had an income of Rs 50,000 or greater. Regarding the number of members of the household, 13.8% had three or fewer members, 31.7% had four members, 34.1% had five members, and 20.2% had six or more members.

**Table 1:** Characteristics of the study population (n = 543).

Variables	Total, n	Proportion, %	Variables	Total, n	Proportion, %	Variables	Total, n	Proportion, %
**Child factors**			**Maternal factors**			**Family factors**		
**Sex**			**Age (years)**			**Ethnicity**		
Boy	204	37.6	20–29	50	9.2	Sinhala	518	95.4
Girl	339	62.4	30–39	284	52.3	Tamil	14	2.6
**Age (years)**			40–49	169	31.1	Muslim	10	1.8
5	43	7.9	50–59	10	1.8	Other	1	0.2
6	118	21.7	Missing	30	5.5	**Religion**		
7	101	18.6	**Educational status**			Buddhist	370	68.1
8	113	20.8	No education	8	1.5	Christian	153	28.2
9	103	19.0	Incomplete primary	12	2.2	Islam	10	1.8
10	65	12.0	Complete primary	73	13.4	Hindu	5	0.9
**Grade**			Incomplete secondary	186	34.3	Other	3	0.6
1	125	23.0	Complete secondary	164	30.2	Missing	2	0.4
2	102	18.8	Higher	70	12.9	**Household income (monthly average, Rs**^a^**)**		
3	116	21.4	Others	2	0.4	< 7,500	22	4.1
4	95	17.5	Missing	28	5.2	7,500–< 15,000	106	19.5
5	105	19.3	**Employment status**			15,000–< 50,000	316	58.2
**Child nutritional status (BAZ)**			Housewife	373	68.7	≥ 50000	61	11.2
Thinness (< -2SD)	105	19.3	Government	27	5.0	Unknown	29	5.3
Normal (-2SD–+1SD)	365	67.2	Private Sector	49	9.0	Missing	9	1.7
Overweight and obese (> +1SD)	73	13.4	Self-employment	44	8.1	**Total number of members in the household**		
Obese (> +2SD)	29	5.3	Agriculture	0	0.0	2	1	0.2
			Schooling	1	0.2	3	74	13.6
			Job-hunting	4	0.7	4	172	31.7
			Unemployment	19	3.5	5	185	34.1
			Other	7	1.3	6	80	14.7
			Missing	19	3.5	≥ 7	30	5.5
			**Maternal nutritional status (BMI, kg/m**^**2**^**)**			Missing	1	0.2
			Thinness (< 18.5)	27	5.0			
			Normal (18.5–< 25)	148	27.3			
			Overweight (25–< 30)	132	24.3			
			Obesity (≥ 30)	66	12.2			
			Missing	170	31.3			

^a^Rs: Sri Lankan Rupee

**Fig 2:**
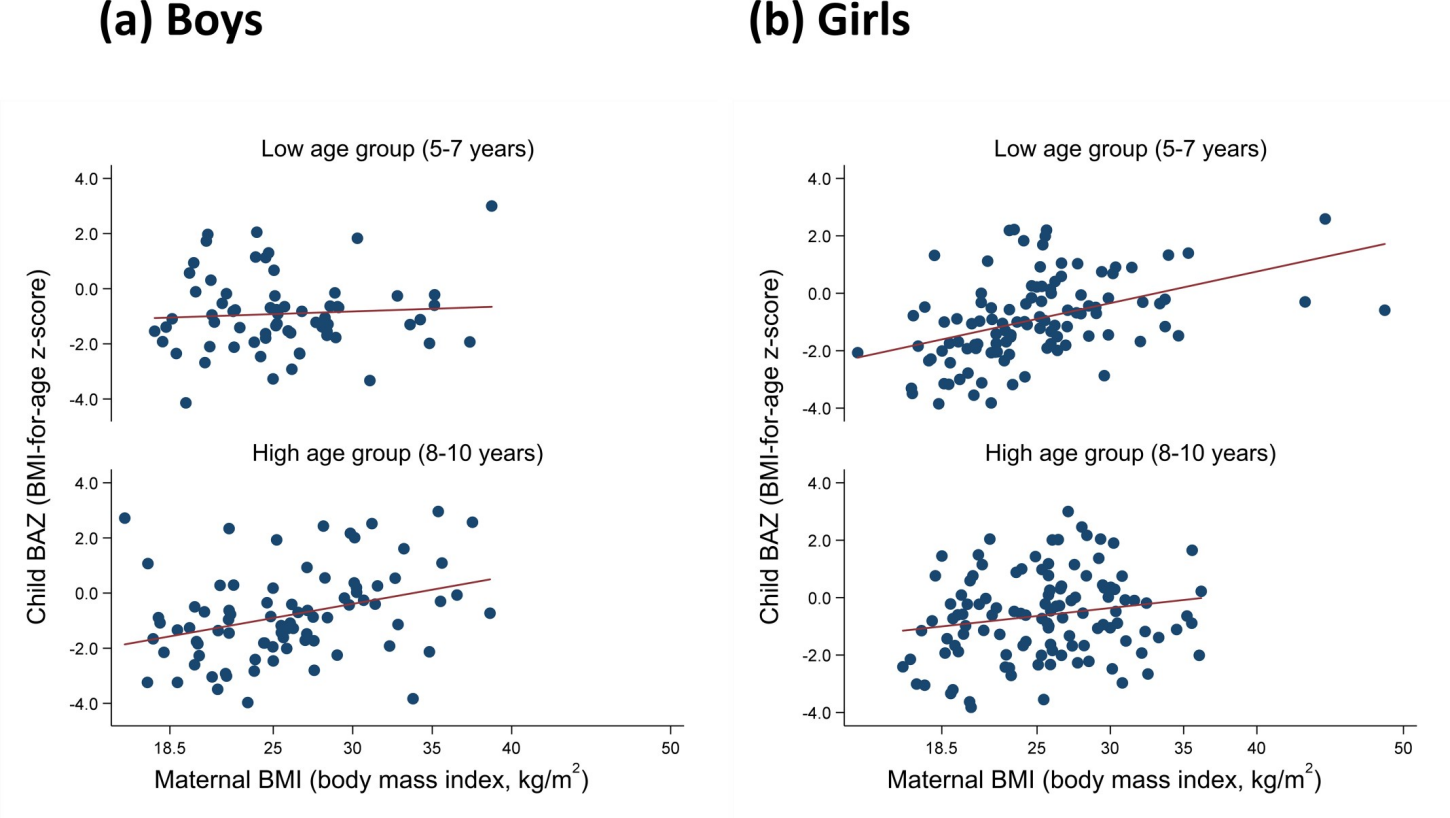
Distribution of maternal body mass index (BMI) and child BMI for age z-score (BAZ) according to sex and age group (low: 5–7 years old, high: 8–10 years old). (A) Boys: Low, *r* = 0.02, *p* = 0.90; High: *r* = 0.39, *p* < 0.001. (B) Girls: Low, *r* = 0.51, *p* < 0.001; High, *r* = 0.18, *p* = 0.06.

Fig 2 shows the distributions of the nutritional status of mother-child pairs (maternal BMI and child BAZ) by sex and age. In boys, there was a statistically significant correlation between maternal BMI and child BAZ in the higher age group (aged 8–10 years) (Spearman’s rank correlation coefficient *r* = 0.39, *p* < 0.001), but this was not observed in the lower age group (aged 5–7 years). Meanwhile, the correlation was significant in girls in the lower age group (*r* = 0.51, *p* < 0.001) but not in the higher age group.

Table 2 shows the distribution of nutritional status among mother-child pairs after excluding ineligible observations (n = 339). The percentages of maternal and child’s nutritional status classified in the same category were 3.2% for thinness, 24.2% for normal, and 8.6% for overweight including obesity. For mothers with high BMI, the proportion of child thinness was 5.3%. In the group where the child’s nutritional status was normal, BAZ showed a significant trend according to maternal nutritional status (*p* < 0.01).

**Table 2:** Distribution of nutritional status among mother-child pairs in urban Sri Lanka (n = 339).

		Mother (BMI)								
Variables		Thinness (< 18.5) n = 25		Normal (18.5–< 25) n = 133		Overweight/obesity (≥ 25) n = 181				
								Obesity (≥ 30) n = 61		
	Total, n	Total, n	Proportion, %	Total, n	Proportion, %	Total, n	Proportion, %	Total, n	Proportion, %	*P*^a^
Child (BAZ)										
Thinness (< -2SD)	65	11	3.2	36	10.6	18	5.3	7	2.1	0.08
Normal (-2SD to +1SD)	228	12	3.5	82	24.2	134	39.5	41	12.1	0.01
Overweight and obesity (> +1SD)	46	2	0.6	15	4.4	29	8.6	13	3.8	0.37
Obesity (> +2SD)	20	1	0.3	5	1.5	14	4.1	6	1.8	0.84

BMI: body mass index, BAZ: BMI-for-age z-score, SD: standard deviation.

The denominator of each proportion is the total number (n = 339).

^a^*p* for trend (maternal thinness to overweight/obesity in each category of child’s nutritional status)

Table 3 shows the results of the trend tests for significant relationships between demographic characteristics and children’s nutritional statuses according to maternal BMI categories (normal or high BMI). Among mothers with normal BMI, a significant relationship was found between maternal employment status and their children’s nutritional status (*p* = 0.016). Thinness was highly prevalent (29.7%) among children of mothers who were housewives, whereas being overweight was highly prevalent (23.1%) among children of mothers who were employed. Among mothers with high BMI, children’s nutritional status was not significantly, but marginally associated with maternal education (*p* = 0.096). Thinness was highly prevalent (22.2%) among children of mothers with low educational status, whereas only 4.4% of the children of mothers with high educational status were in the thinness category.

**Table 3:** Demographic characteristics with nutritional status among mother-child pairs in urban Sri Lanka (n = 314).

Variables	Mothers with normal BMI (n = 133)					Mothers with high BMI (n = 181)				
	Total, n	Thinness, %	Normal, %	Overweight, %	*p*	Total, n	Thinness, %	Normal, %	Overweight, %	*p*
N	133	36	82	15		181	18	134	29	
%		27.1	61.7	11.3		9.9	74.0	16.0	
**Sex**					0.76					0.66
Boy	51	27.5	58.8	13.7		74	10.8	74.3	14.9	
Girl	82	26.8	63.4	9.8		107	9.4	73.8	16.8	
**Grade**					0.55					0.33
1	38	26.3	63.2	10.5		40	7.5	82.5	10.0	
2	26	23.1	57.7	19.2		27	7.4	74.1	18.5	
3	25	32.0	56.0	12.0		42	7.1	69.1	23.8	
4	21	28.6	61.9	9.5		36	8.3	72.2	19.4	
5	23	26.1	69.6	4.4		36	19.4	72.2	8.3	
**Mother’s age (years)**					0.32					0.81
20–29	18	11.1	66.7	22.2		18	5.6	83.3	11.1	
30–39	78	30.8	60.3	9.0		98	10.2	72.5	17.4	
40–49	34	29.4	58.8	11.8		62	9.7	75.8	14.5	
50–59	3	0.0	100.0	0		3	33.3	33.3	33.3	
**Maternal education**					0.39					0.10
Low (up to primary)	20	45.0	50.0	5.0		27	22.2	59.3	18.5	
Middle (incomplete secondary)	36	19.4	66.7	13.9		63	12.7	73.0	14.3	
High (complete secondary or higher)	77	26.0	62.3	11.7		91	4.4	79.1	16.5	
**Maternal employment status**					0.02					0.82
Housewife	101	29.7	63.4		6.9	132	11.4	71.2	17.4	
Employment	26	19.2	57.7	23.1		41	7.3	85.4	7.3	
Others	6	16.7	50.0	33.3		8	0.0	62.5	37.5	
**Household income (monthly average, Rs.)**						0.88					0.18
Low (< 15,000)	38	31.6	52.6	15.8		37	8.1	83.8	8.1	
Middle (15,000–< 50,000)	84	25.0	66.7	8.3		116	11.2	72.4	16.4	
High (≥ 50,000)	11	27.3	54.6	18.2		28	7.1	67.9	25.0	
**Total number of members of the household**						0.08					0.32
2–3	22	27.3	50.0	22.7		22	4.6	68.2	27.3	
4	44	15.9	75.0	9.1		70	11.4	71.4	17.1	
5	41	31.7	61.0	7.3		58	10.3	81.0	8.6	
6–10	26	38.5	50.0	11.5		31	9.7	71.0	19.4	

BMI: body mass index, Mothers with normal BMI: 18.5–< 25 kg/m^2^, Mothers with high BMI: > 25 kg/m^2^, Child BAZ: BMI-for-age z-score, Thinness: BAZ < -2SD, Normal: BAZ -2SD–+1SD, Overweight: BAZ > +1SD, SD: standard deviation

Table 4 presents the aOR for nutritional status among children computed using multiple logistic regression models with backward stepwise selection. The factors were analyzed separately for child thinness (compared to normal) and child overweight and obesity (compared to normal). For child thinness, variables of maternal educational status, maternal employment status, and maternal nutritional status remained after applying multiple logistic regression models with backward stepwise selection. Low maternal education (aOR = 2.33, 95% CI: 1.08–5.00, *p* < 0.05) was significantly associated with child thinness compared to high maternal education. Mothers with overweight and obesity (aOR = 0.30, 95% CI: 0.16–0.58, *p* < 0.001) were less likely to have a child with thinness compared to mothers with normal BMI. For child overweight and obesity, variables of maternal employment status and HEI remained after applying multiple logistic regression models with backward stepwise selection. Mothers who selected “others” as their employment status (compared to housewives), and those with low and middle HEI (compared to high HEI), were not significantly, but marginally associated with a child being overweight (including obese).

**Table 4:** Adjusted odds ratios (aOR) for child nutritional status in urban Sri Lanka, using crude analysis and multiple regression models (n = 339).

Variables	Child thinness (n = 293)						Child overweight and obesity (n = 274)			
	Model 1^a^			Model 2^b^			Model 1^a^		Model 2^b^	
	OR	(95% CI)		aOR	(95% CI)		OR	(95% CI)	aOR	(95% CI)
**Sex**										
Boy	0.82	(0.46–1.46)					1.16	(0.61–2.20)		
Girl	1.00						1.00			
**Maternal education**										
Low (up to primary)	2.44	(1.19–5.01)	*		2.33	(1.08–5.00)	*	1.04	(0.39–2.77)		
Middle (incomplete secondary)	1.12	(0.59–2.14)		1.18	(0.60–2.33)		1.14	(0.57–2.29)		
High (complete secondary or higher)	1.00		1.00				1.00			
**Maternal employment status**											
Housewife	1.00			1.00			1.00		1.00	
Employment	0.63	(0.31–1.29)		0.64	(0.30–1.39)		0.85	(0.38–1.90)	0.72	(0.31–1.68)
Others	0.31	(0.04–2.47)		0.25	(0.03–2.16)		2.56	(0.82–8.00)	2.44	(0.77–7.72)
**Household equivalent income (HEI, monthly average, Rs.)**											
Low (< 15,000)	1.75	(0.59–5.20)						0.56	(0.20–1.51)	0.48	(0.17–1.37)
Middle (15,000–< 50,000)	1.47	(0.53–4.07)					0.53	(0.22–1.27)	0.48	(0.19–1.20)
High (≥ 50,000)	1.00							1.00		1.00	
**Maternal nutritional status (BMI, kg/m**^**2**^**)**										
Thinness (< 18.5)	2.09	(0.84–5.17)		2.22	(0.86–5.75)		0.91	(0.18–4.49)		
Normal (18.5–< 25)	1.00			1.00			1.00			
Overweight and obesity (≥ 25)	0.31	(0.16–0.57)	***	0.30	(0.16–0.58)	***	1.18	(0.60–2.34)		

*** *p* < 0.001

* *p* < 0.05, Rs.: Sri Lankan Rupee, BMI: body mass index, aOR: adjusted odds ratio; 95% CI: 95% confidence interval

^a^Model 1: Crude analysis. Each variable was separately entered into the model. Household equivalent income (HEI) was calculated as household income divided by the square root of the number of people per household.

^b^Model 2: For multiple regression, child sex, maternal education, maternal employment status, HEI, maternal age, and maternal nutritional status were forced into the model, and selected by backward stepwise selection with a 0.35 of significant level of removal from the model. Only the selected variables were used for calculating aOR.

## Discussion

In the present sample of urban Sri Lankan primary school students, the percentage of thinness and overweight (including obesity) was 19.3% and 13.4%, respectively. In other words, approximately one in three students suffered from malnutrition. Statistically significant positive correlations were found between maternal BMI and child BAZ in younger boys and older girls. The multiple logistic regression analysis with backward stepwise selection showed that mothers with lower education were significantly association with child thinness compared to mothers with higher education. Additionally, mothers with high BMI were less likely to have a child with thinness compared to mothers with normal BMI. In child overweight and obesity, no significant association was observed but, mothers whose employment status was “others” were marginally more likely to have an overweight and obese child compared to housewives. Also, children of mothers with lower and middle HEI had a marginally higher association with being overweight and obese as compared to children of those with higher HEI.

The percentages of overweight and obese children in the present sample was greater than those reported in the Annual Health Bulletin in 2015, suggesting that over-nutrition in urban areas may be increasing. This finding coincides with the results of some studies conducted in China and Brazil [13]. The fact of co-existing of under- and over-nutrition among children within a community suggests the need for further research on potential underlying environmental factors, such as school lifestyles.

In the present study, the percentage of maternal overweight/obesity was 36.5%, which was nearly three times that of the percentage among children. Not only were there cases of mothers and their children having the same nutritional status (both being underweight or overweight) but there were also cases where the child was under-nourished (thinness) while the mother was over-nourished (overweight/obesity). In other words, the double burden of malnutrition was observed within the household. Given that parental recognition of weight problems in their children is unreliable [14], there can be a gap between parental perception and their child’s actual weight status. Consequently, it may be difficult for parents to monitor their child’s physical development accurately. This finding suggests the need for schools to monitor students’ physical development through, for example, regular check-ups, and to provide parents with nutrition education. Generally, the mother prepares the family’s daily meals, and her preferences on the quantity and taste of the food she prepares may be unconsciously influenced by her nutritional knowledge and physical status, which could bias the child’s calorie intake. More detailed studies may be needed to understand how healthy dietary environments could be created and how meal patterns could be established to enable families to follow nutritionally balanced eating habits that ensure healthy growth.

The correlation between the nutritional statuses of mothers and their children differed by gender and age. Regarding the association between maternal BMI and child BAZ, statistically significant positive correlations were found in older boys (*r* = 0.39, *p* < 0.001) and younger girls (*r* = 0.51, *p* < 0.001) (Fig 2). There may be sex-related developmental differences that result in different periods of particular susceptibility to influences from maternal physical status. This may have been because girls’ physical development changes significantly as the body tends to store fat at the onset of menstruation in later childhood. Some studies have reported that the risk of lifestyle-related diseases in later life differs depending on the age at which adiposity rebound occurred in childhood [15]. Another study showed that irregular menstruation is frequent in obese adolescent Sri Lankan girls (aged 14–18 years) [16]. Therefore, in the prevention of risks related to obesity and lifestyle diseases in adulthood, various biological factors and the process of physical development during childhood need to be considered. Taking a life-course approach, because a positive correlation was found between maternal BMI and child BAZ in both boys and girls (albeit at different ages), nutrition-related interventions in school-age children may be important as a promising way to interrupt malnutrition’s negative chain of events in later life.

Lower maternal education was associated with child thinness as compared to higher maternal education. A study in Mexico found that very young children experiencing the double burden of concurrent overweight and stunting had mothers with lower education [8]. Meanwhile, our study suggests that the double burden of concurrent maternal overweight and child thinness was more prevalent in mothers with lower education. Evidence indicates that the association between education and the double burden of malnutrition is not exclusive within the same generation and that the shared environment of nutritional imbalances within the same household may play an important role besides genetic factors [17].

In mothers with high BMI, the proportion of overweight children was lower in low and middle HEI households as compared to high HEI households. Obesity was more common among Sri Lankan adolescent girls (aged 14–18 years) from high-income families [16]. Households within wealthier quintiles were more likely to have double burden of malnutrition among mothers and their children aged 6–59 months in rural Indonesia and Bangladesh [18]. Our findings suggest that socioeconomic factors may be important predictors of being overweight throughout life, not only in later childhood and adolescence but also in adulthood, though further studies are required.

Mothers who selected “others” as their employment status, such as job-hunting and unemployment, had a higher percentage of children with overweight and obesity compared to housewives. A UK birth cohort study showed that maternal employment status was associated with overweight in children [11]. Mothers who need to concentrate on other things may not be able to spend adequate time on child-raising, such as preparing healthy meals and picking children up from school. Particularly in urban areas, it is possible that children eat high-fat junk food instead of healthy meals, and they may have fewer opportunities for exercise. While the present results suggest that differences in children’s nutritional statuses may be due to socioeconomic factors, it would be difficult to customize nutritional support to every family’s circumstances. It may be more effective and fairer to consider how to implement universal strategies in schools to help improve children’s nutritional status.

This study had several limitations. First, we recruited students on a school basis rather than a community basis. A large number of missing values for maternal BMI were observed (n = 170), however, the systematic error due to the missing values would not affect the final results since there was no difference in the relationship between the nutritional status among children and SES with or without missing data. Second, we used the WHO’s growth reference [12] rather than standards specific to Sri Lankans, owing to the absence of a more reliable and suitable standard for Sri Lankans based on their racial differences in body type. Also, the WHO growth reference is easily accessible and it allowed us to conduct international comparisons. In resource-limited environments such as developing countries, the WHO reference could be useful to objectively describe a population’s nutritional status, because it is relatively easy to gather the data (age, height, and weight) needed to compute child BAZ. Growth charts not only enable the monitoring of child growth longitudinally, but they can be used to evaluate the distribution of nutritional statuses in populations cross-sectionally [19]. While these limitations need to be considered when interpreting the results, this study revealed the associations between socioeconomic factors and the double burden of malnutrition among mothers and their primary school-aged children in urban Sri Lanka.

## Conclusions

This study showed that, in a sample of urban Sri Lankan primary school children, the positive correlation between nutritional status in mothers and their children differed by sex and age. Furthermore, the results highlighted a double burden of malnutrition within households, with co-existence of child thinness and maternal overweight and obesity. Maternal education might be associated with child thinness. These findings suggested that nutritional assessments, such as regular growth monitoring for children in schools and support for creating an appropriate dietary environment in households that is accessible for the socially vulnerable, are necessary for improving the health of mothers and their children.

## Supporting information

S1 File Survey questionnaire (in Sinhala). (PDF)

S2 File Survey questionnaire (translated in English). (PDF)
