# Correction: Double burden of maternal and child malnutrition and socioeconomic status in urban Sri Lanka

**DOI:** 10.1371/journal.pone.0230785

**Published:** 2020-03-19

**Authors:** Chisa Shinsugi, Deepa Gunasekara, N. K. Gunawardena, Wasanthi Subasinghe, Miki Miyoshi, Satoshi Kaneko, Hidemi Takimoto

There are formatting errors in Tables 3 and 4. In addition, there is an error in the caption for Table 3. Please see the correct tables and captions here.

**Table 3:** 

Variables	Mothers with normal BMI (n = 133)					Mothers with high BMI (n = 181)				
	Total, n	Thinness, %	Normal, %	Overweight, %	*p*	Total, n	Thinness, %	Normal, %	Overweight, %	*p*
N	133	36	82	15		181	18	134	29	
%		27.1	61.7	11.3			9.9	74.0	16.0	
**Sex**					0.76					0.66
Boy	51	27.5	58.8	13.7		74	10.8	74.3	14.9	
Girl	82	26.8	63.4	9.8		107	9.4	73.8	16.8	
**Grade**					0.55					0.33
1	38	26.3	63.2	10.5		40	7.5	82.5	10.0	
2	26	23.1	57.7	19.2		27	7.4	74.1	18.5	
3	25	32.0	56.0	12.0		42	7.1	69.1	23.8	
4	21	28.6	61.9	9.5		36	8.3	72.2	19.4	
5	23	26.1	69.6	4.4		36	19.4	72.2	8.3	
**Mother’s age (years)**					0.32					0.81
20–29	18	11.1	66.7	22.2		18	5.6	83.3	11.1	
30–39	78	30.8	60.3	9.0		98	10.2	72.5	17.4	
40–49	34	29.4	58.8	11.8		62	9.7	75.8	14.5	
50–59	3	0.0	100.0	0		3	33.3	33.3	33.3	
**Maternal education**					0.39					0.10
Low (up to primary)	20	45.0	50.0	5.0		27	22.2	59.3	18.5	
Middle (incomplete secondary)	36	19.4	66.7	13.9		63	12.7	73.0	14.3	
High (complete secondary or higher)	77	26.0	62.3	11.7		91	4.4	79.1	16.5	
**Maternal employment status**					0.02					0.82
Housewife	101	29.7	63.4	6.9		132	11.4	71.2	17.4	
Employment	26	19.2	57.7	23.1		41	7.3	85.4	7.3	
Others	6	16.7	50.0	33.3		8	0.0	62.5	37.5	
**Household income (monthly average, Rs.)**					0.88					0.18
Low (< 15,000)	38	31.6	52.6	15.8		37	8.1	83.8	8.1	
Middle (15,000–< 50,000)	84	25.0	66.7	8.3		116	11.2	72.4	16.4	
High (≥ 50,000)	11	27.3	54.6	18.2		28	7.1	67.9	25.0	
**Total number of members of the household**					0.08					0.32
2–3	22	27.3	50.0	22.7		22	4.6	68.2	27.3	
4	44	15.9	75.0	9.1		70	11.4	71.4	17.1	
5	41	31.7	61.0	7.3		58	10.3	81.0	8.6	
6–10	26	38.5	50.0	11.5		31	9.7	71.0	19.4	

BMI: body mass index, Mothers with normal BMI: 18.5–< 25 kg/m^2^, Mothers with high BMI: ≥ 25 kg/m^2^, Child BAZ: BMI-for-age z-score, Thinness: BAZ < -2SD, Normal: BAZ -2SD–+1SD, Overweight: BAZ > +1SD, SD: standard deviation

**Table 4:** 

Variables	Child thinness (n = 293)				Child overweight and obesity (n = 274)			
	Model 1^a^		Model 2^b^		Model 1^a^		Model 2^b^	
	OR	(95% CI)	aOR	(95% CI)	OR	(95% CI)	aOR	(95% CI)
**Sex**								
Boy	0.82	(0.46–1.46)			1.16	(0.61–2.20)		
Girl	1.00				1.00			
**Maternal education**								
Low (up to primary)	2.44	(1.19–5.01)*	2.33	(1.08–5.00)*	1.04	(0.39–2.77)		
Middle (incomplete secondary)	1.12	(0.59–2.14)	1.18	(0.60–2.33)	1.14	(0.57–2.29)		
High (complete secondary or higher)	1.00		1.00		1.00			
**Maternal employment status**								
Housewife	1.00		1.00		1.00		1.00	
Employment	0.63	(0.31–1.29)	0.64	(0.30–1.39)	0.85	(0.38–1.90)	0.72	(0.31–1.68)
Others	0.31	(0.04–2.47)	0.25	(0.03–2.16)	2.56	(0.82–8.00)	2.44	(0.77–7.72)
**Household equivalent income (HEI, monthly average, Rs.)**								
Low (< 15,000)	1.75	(0.59–5.20)			0.56	(0.20–1.51)	0.48	(0.17–1.37)
Middle (15,000–< 50,000)	1.47	(0.53–4.07)			0.53	(0.22–1.27)	0.48	(0.19–1.20)
High (≥ 50,000)	1.00				1.00		1.00	
**Maternal nutritional status (BMI, kg/m**^**2**^**)**								
Thinness (< 18.5)	2.09	(0.84–5.17)	2.22	(0.86–5.75)	0.91	(0.18–4.49)		
Normal (18.5–< 25)	1.00		1.00		1.00			
Overweight and obesity (≥ 25)	0.31	(0.16–0.57)***	0.30	(0.16–0.58)***	1.18	(0.60–2.34)		

*** *p* < 0.001,

* *p* < 0.05,

Rs.: Sri Lankan Rupee, BMI: body mass index, aOR: adjusted odds ratio; 95% CI: 95% confidence interval

^a^Model 1: Crude analysis. Each variable was separately entered into the model. Household equivalent income (HEI) was calculated as household income divided by the square root of the number of people per household.

^b^Model 2: For multiple regression, child sex, maternal education, maternal employment status, HEI, maternal age, and maternal nutritional status were forced into the model, and selected by backward stepwise selection with a 0.35 of significant level of removal from the model. Only the selected variables were used for calculating aOR.

## References

[1] Shinsugi C, Gunasekara D, Gunawardena NK, Subasinghe W, Miyoshi M, Kaneko S, et al (2019) Double burden of maternal and child malnutrition and socioeconomic status in urban Sri Lanka. PLoS ONE 14(10): e0224222 10.1371/journal.pone.0224222 31639148 PMC6805006

