# Supplementary material for: Double burden of maternal and child malnutrition and socioeconomic status in urban Sri Lanka
Source: PLoS One. 2019 Oct 22;14(10):e0224222. doi: 10.1371/journal.pone.0224222 (PMC6805006; doi:10.1371/journal.pone.0224222)
Supplement: S1 File — (PDF) [file pone.0224222.s001.pdf]

Serial no:				
School Name				
Type	1AB	1C	2	3

Grade		Class		Date	/ Sep /2017

දත්ත එකතු කිරීමේ පත්‍රය

ගම්පහ දිස්ත්‍රික්කයේ ප්‍රාථමික පාසල් දරුවන් අතර මන්දපෝෂණය පිළිබඳ අධ්‍යයනයක්

ළමයාගේ මානවමිතික මිනුම්
1. අනු අංකය : ________________   2. උස(cm) : ____________ cm   3. බර (kg) : ____________ kg   4. ඉණ වටය (cm) : ____________ cm   5. ඉහල බාහු මධ්‍ය වටප්‍රමාණය (mm) : ____________ mm   6. සමේ ඝණකම(mm) :   6-1. Triceps : ______________ mm   6-2. Biceps : ___________ mm   6-3. Subscapular : ___________ mm   6-4. Suprailiac : _______________ mm

ප්‍රාථමික පාසල් දරුවන් අතර මන්දපෝෂණයට සම්බන්ධ අවදානම් සාධක ගැන අපි සොයා බලමු. කරුණාකර පහත සඳහන් ප්‍රශ්න වලට ඔබේ දැනුමෙන් පිළිතුරු ලබා දෙන්න.

මතක තබාගන්න:

1. මෙය පරීක්ෂණයක් නොවේ. නිවැරදි හා වැරදි පිළිතුරු නොමැත.
2. කරුණාකර ඔබ අවංකව සහ නිවැරදිව සියලු ප්‍රශ්න වලට පිළිතුරු දෙන්න මෙය ඉතා වැදගත්
3. කරුණාකර ඔබේ පිළිතුරේ තේරුම සහිත තීරුව සම්පූර්ණයෙන්ම පිරවීම සඳහා පැන්සලක් භාවිතා කරන්න.
4. පිළිතුරු සැපයීමේදී පහත සඳහන් උපදෙස් භාවිතා කරන්න.

   කොටුවේ හරි ලකුණ (✔) දමන්න   උදා: ✔ සිංහල
   සුදුසු අංකය රවුම් (◯) කරන්න   උදා.: 1 ② 3 4
   නිශ්චිත උපදෙස් නොමැති නම් ඔබේ පිළිතුර ලියන්න.

5. මෙම ප්‍රශ්නාවලියෙහි "ළමා සෞඛ්‍ය තත්ත්වය" කොටස පිරවීමේදී "ළමා සෞඛ්‍ය සංවර්ධන වාර්තාව" වෙත යොමුවන්න.

ප්‍රශ්නාවලිය

දරුවාගේ මූලික තොරතුරු	
1. නම (වාසගම/ මුල් නම) :	
2. **1** □ස්ත්‍රී **2** □පුරුෂ	3. උපන් දිනය(දින/මාස/අවු):_____/____/_____<br>වයස (දින/මාස/අවු):_____/____/_____(වයස අදාල දිනයට)
4. ජාතිය : **1** □සිංහල **2** □දෙමළ **3** □මුස්ලිම් **4** □වෙනත්	5. භාෂාව : **1** □සිංහල **2** □දෙමළ **3** □ඉංග්‍රීසි **4** □වෙනත්
6. ආගම : **1** □බෞද්ධ **2** □හින්දු **3** □ඉස්ලාම් **4** □ ක්‍රිස්තියානි **5** □වෙනත්	

දරුවාගේ පවුලේ විස්තර

7. විස්තර සපයන්නා **1** □මව **2** □පියා **3** □භාරකරු **4** □වෙනත් ( )

8. පවුලේ සාමාජිකයින් ගණන? 1 මුළු ගණන (2 වැඩිහිටි - 3 ළමා- * (දරුවා හැර)

විස්තර : **1** □සීයා (ගණන) **2** □ආච්චි (ගණන) **3** □පියා **4** □මව **5** □වැඩිමහල් සහෝදරයින් (ගණන)

6 □වැඩිමහල් සහෝදරියන් (ගණන) **7** □බාල සහෝදරයින් (ගණන) **8** □බාල සහෝදරියන් (ගණන)

9 □වෙනත්: (____________________)

9. පවුලේ මාසික ආදායම?

1 □< Rs.7,500 **2** □Rs. 7,500 – < Rs,15,000 3 □Rs. 15,000 – < Rs.50,000 4 □ Rs. 50,000 වැඩි 5 □ නොදනී

10. කරුණාකර දරුවාගේ පවුලේ ආකෘතියට පිළිතුරු දෙන්න (මෙම පවුල යනු දරුවාගේ දෙමව්පියන් සහ සහෝදර සහෝදරියන්).

පවුල් ව්‍යුහය	උදාහරණ	a	b	c	d	e	f
A) සම්බන්ධතාවය<br>**1** මව **2** පියා **3** වැඩිමහල් සොයුරා **4** වැඩිමහල් සොයුරිය **5** බාල සොයුරා **6** බාල සොයුරිය **7** වෙනත්	1						
B) වයස (අවුරුදු)	40						
C) උස සහ බර	170 cm<br>70 kg	___ cm<br>___ kg	___ cm<br>___ kg	___ cm<br>___ kg	___ cm<br>___ kg	___ cm<br>___ kg	___ cm<br>___ kg
D) අධ්‍යාපන තත්ත්වය<br>*එකක් තෝරන්න<br>1. අධ්‍යාපනය නැත<br>2. අසම්පූර්ණ ප්‍රාථමික<br>3. සම්පූර්ණ ප්‍රාථමික<br>4. අසම්පූර්ණ ද්විතික<br>5. සම්පූර්ණ ද්විතික<br>6. උසස් 7. වෙනත්	1 2 ③<br>4 5 6<br>7	1 2 3<br>4 5 6<br>7	1 2 3<br>4 5 6<br>7	1 2 3<br>4 5 6<br>7	1 2 3<br>4 5 6<br>7	1 2 3<br>4 5 6<br>7	1 2 3<br>4 5 6<br>7

E) වෘත්තීය මට්ටම<br>* ප්‍රධාන කාර්යයක් තෝරන්න<br>1. ළදරු 2. ශිෂ්‍ය<br>3. රැකියාවක් බලාපොරොත්තුවෙන් සිටීම<br>4. රැකියාවක් නොමැත<br>5. කෘෂිකර්මාන්තය<br>6. රජයේ අංශය<br>7. පුද්ගලික අංශය<br>8. ව්‍යාපාරික / ස්වයං රැකියාවක නියුක්ත වන<br>9. ගෘහණියක්<br>10. අනෙක්	1 2 3<br>4 5 ⑥<br>7 8 9<br>10	1 2 3<br>4 5 6<br>7 8 9<br>10	1 2 3<br>4 5 6<br>7 8 9<br>10	1 2 3<br>4 5 6<br>7 8 9<br>10	1 2 3<br>4 5 6<br>7 8 9<br>10	1 2 3<br>4 5 6<br>7 8 9<br>10	1 2 3<br>4 5 6<br>7 8 9<br>10
F) සෞඛ්‍ය තත්ත්වය<br>* අදාළ වන සියල්ල සටහන් කරන්න<br>1. දියවැඩියාව 2. වකුගඩු රෝග<br>3. ඩෙංගු (පසුගිය මාස 3 තුල)<br>4. වෙනත් (සඳහන් කරන්න)	1 2 ③<br>4<br>________	1 2 3<br>4<br>________	1 2 3<br>4<br>________	1 2 3<br>4<br>________	1 2 3<br>4<br>________	1 2 3<br>4<br>________	1 2 3<br>4<br>________

දරුවාගේ සෞඛ්‍ය තත්ත්වය

11. ඔබ සතුව "ළමා සෞඛ්‍ය වර්ධන සටහන : 1 □ ඇත 2 □ නැත

12. උපත් බර : 1 □ <2,500g 2 □2,500g ත් 4,000g අතර 3 □ >4,000g 4 □ නොදනී

13. ප්‍රතිශක්තීකරණ තොරතුරු (අදාළ සියල්ල ලකුණු කරන්න)

උපතින් පසු	සති 0-4	මාස 2	මාස 4	මාස 6	මාස 9	මාස 12	මාස 18	අවුරුදු 3	අවුරුදු 5
එන්නත	1☐BCG	1☐OPV1<br>2☐DTwP1<br>3☐HepE1<br>4☐Hib1	1☐OPV2<br>2☐DTwP2<br>3☐HepE2<br>4☐Hib2<br>5☐IPV	1☐OPV3<br>2☐DTwP3<br>3☐HepE3<br>4☐Hib3	1☐LAJEV	1☐MMR1	1☐OPV4<br>2☐DTwP4	1☐MMR2	1☐OPV5<br>2☐DT1

14. මෙම රෝග වැළදී තිබේද; (අදාළ සියල්ල ලකුණු කරන්න)
1 □ නිව්මෝනියාව 2 □ ඇදුම 3 □ මැලේරියාව 4 □ ඉන්ෆ්ලුවන්සාව 5 □ රක්තහීනතාවය
6 □ පණු රෝග 7 □ දියවැඩියාව 8 □ ඩෙංගු 9 □ වෙනත්: (____________)

15. මෑත කාලීන රෝගී තත්ත්වයන් (පසුගිය මාසය ඇතුළත) අදාළ සියල්ල ලකුණු කරන්න
1 □ පාචනය 2 □ වමනය 3 □ රක්තහීනතාවය 4 □ හිසරදය 5 □ උණ
6 □ බර අඩුවීම 7 □ ආසාදිත රෝග 8 □ මාර්ග අනතුරු 9 □ වෙනත්: (____________)

16. දරුවා සාමාන්‍යයෙන් අවදිවන හෝ නින්දට යන වේලාව?: 1 නින්දට යාම (ප.ව :) 2 අවදිවීම (පෙ.ව :)

17. නිවසේ සිට පාසලට යාමට ගතවන කාලය? එකක් සඳහා තෝරන්න
1 □ පයින් 2 □බයිසිකලයෙන් 3 □ කාරයෙන් 4 □පාසල් බසයෙන්/වෑන් 5 □ දුම්රියෙන් 6 □ වෙනත් ( )
1 □ < මිනිත්තු 10 2 □ මිනිත්තු 10 - < 30 3 □ මිනිත්තු 30 - < 60 4 □ ≥ මිනිත්තු 60

18. පාසල් ශාරීරික අධ්‍යාපන කාලපරිච්ඡේදයේ හැර දරුවා ශාරීරික අභ්‍යාස වල නිරත වේද? (සමාජ ක්‍රියාකාරකම්, ක්‍රීඩා සමාජ ආදිය)

1 □ නැත **2** □ ඔව් ◊ එසේ නම්, කරුණාකර ක්‍රියාකාරකම විස්තර කරන්න: ____________________________

19. දරුවාගේ ශාරීරික ක්‍රියාකාරිත්වය: විවේක වේලාවන්හිදී කරන ක්‍රියාකාරකම් වල වාරගණන සහ සාමාන්‍ය සතියක එක් ක්‍රියාකාරකමක් සඳහා ගත වන කාලය

ශාරීරික ක්‍රියාකාරකම්	වාර ගණන (සතියකට)					එක් අවස්ථාවක වැයකළ කාලය				
	හැමදාම වගේ	5-6 වාරයක්	3-4 වාරයක්	1-2 වාරයක්	කවදාත් නැත	විනාඩි 30 කට අඩු කාලයක්	30-59 විනාඩි	1-2 පැය	2-3 පැය	පැය 3 කට වඩා
a) බලගතු-තීව්‍රතා ක්‍රියාකාරකම් (උදා: ධාවනය සහ පිහිනීම)	1	2	3	4	5	a	b	c	d	e
b) මධ්‍යස්ථ-තීව්‍රතා ක්‍රියාකාරකම් (උදා: ඉක්මන් ඇවිදීම හා නැටුම්)	1	2	3	4	5	a	b	c	d	e
ඇ) සැහැල්ලු තීව්‍රතා ක්‍රියාකාරකම් (උදා: සෙමින් ඇවිද)	1	2	3	4	5	a	b	c	d	e

20. ව්‍යායාමෙන් තොර හැසිරීම: සාමාන්‍යයෙන් දරුවා වාඩි වී හෝ වැතිරී සිටින්නේ කොපමණ කාලයක්ද ?: දිනකට පැය _______________

දරුවාගේ හැසිරීම (ප්‍රබලතා සහ දුබලතා)			
21. සෑම අයිතමයක් සඳහාම කරුණාකර කොටුව සලකුණු කරන්න (සත්‍ය වශයෙන්ම, යම් දුරකට සත්‍ය හෝ නියත සත්‍යයක් නොවේ). ඔබට සියලු අයිතම වලට පිළිතුරු ලබා ගත හැකි නම් එය අපට උපකාර වනු ඇත! පසුගිය මාස හය තුළ ඔබේ දරුවාගේ හැසිරීම පදනම් කරගෙන ඔබේ පිළිතුරු දෙන්න. (එක් එක් සඳහා එකක් තෝරන්න)	**වැරදිය**	බොහෝවිට නිවාරදිය	**ඉතා නිවැරදිය**
A. අන් අයගේ හැඟීම් ගැන සිතා බලයි.	1	2	3
B. නොසන්සුන්, අධික්‍රියාකාරී, දිගු කලක් රැඳී සිටීමට නොහැකිය	1	2	3
C. හිසරදය, බඩේ වේදනාව හෝ අසනීප වලට ගොදුරු වේ	1	2	3
D. ආහාර, සෙල්ලම් බඩු ආදිය අනෙක් දරුවන් සමග බෙදාගනී	1	2	3
E. නිතර නිතර ඉක්මනින් කේන්ති යයි.	1	2	3
F. සාමාන්‍යයෙන් හුදකලාව සිටීමට, තනිව සෙල්ලම් කිීරීමට කැමැත්තක් දක්වයි	1	2	3
G. සාමාන්‍යයෙන් කීකරුවේ. වැඩිහිටියන්ට අවනත වේ.	1	2	3
H. නිතර කණස්සල්ලෙන් සිටී.	1	2	3
I. තවකෙකු දුකට, කරදරයට පත් වූ විට උදව් කරයි	1	2	3
J. නිතර නොසන්සුන්ය	1	2	3
K. එක හොද යහළුවෙක් හෝ සිටීයි	1	2	3
L. නිතරම අනෙක් ළමුන් සමග රණ්ඩු කරයි හෝ බාධා කරයි.	1	2	3
M. නිතරම දුකින් සිටියි, මානසික අවපීඩනයෙන් සිටියි.	1	2	3

N. සාමාන්‍යයෙන් අනෙක් ළමුන්ගේ කැමැත්ත දිනා සිටියි	1	2	3
O. සිත එක දෙයකට යොමු කිරීමට නොහැකිය.	1	2	3
P. අලුත් අවස්ථාවකට මුහුණ දීමේදී කළබල වෙයි, ආත්ම විශ්වාසය බිඳවැටෙයි.	1	2	3
Q. වයසින් අඩු දරුවන්ට කාරුණික වෙයි.	1	2	3
R. නිතරම බොරු කියයි හෝ වංචා කරයි.	1	2	3
S. අනෙකුත් ළමුන්ගෙන් නිතර බාධා ඇති වේ.	1	2	3
T. ස්වේච්ඡාවෙන් අනෙකාට උදව් කිරීමට ඉදිරිපත් වෙයි. (දෙමව්පියන්, ගුරුවරු, අනෙක් ළමුන්)	1	2	3
U. දෙයක් කිරීමට පෙර ඒ ගැන සිතයි.	1	2	3
V. ගෙදරින්, පාසලින්, අනෙක් ස්ථාන වලින් සොරකම් කරයි.	1	2	3
W. වැඩිහිටියන් සමග අනෙක් දරුවන් සමග මිතුරු වනවාට වඩා ඉක්මනින් මිතුරුවෙයි.	1	2	3
X. නිතර බිය වෙයි.	1	2	3
Y. අවසාන වන තෙක් එක් අරමුණකින් ක්‍රියාකරයි.	1	2	3

දරුවාගේ ආහාර ගැනීමේ රටාව

22. ඔබේ දරුවා සාමාන්‍යයෙන් උදේ ආහාරය ගන්නවාද? : **1** □ ඔව් **2** □ නැත

23. පසුගිය සතියේ ඔබේ දරුවා පහත සඳහන් ආහාර අනුභව කළාද?

	**සෑම දිනම**	**5-6 වාරයක්**	**3-4 වතාවක්**	**1-2 වතාවක්**	**කවදාවත් නැත**	**නොදනී**
a) බත් සහ අනෙකුත් ධාන්‍ය වර්ග (මුං ඇට.)	1	2	3	4	5	6
b) අල වර්ග (අල,බතල, මඤ්ඤොක්කා)	1	2	3	4	5	6
c) පාන් (පරාටා / රොටී)	1	2	3	4	5	6
d) මාෂ බෝග / පරිප්පු	1	2	3	4	5	6
e) මාළු	1	2	3	4	5	6
f) මස් (හරක්/ඌරු/කුකුල් මස්)	1	2	3	4	5	6
g) බිත්තර	1	2	3	4	5	6
h) කිරි නිෂ්පාදන (මුදවපු කිරි/දියර කිරි/කිරිපිටි)	1	2	3	4	5	6
i) පොල් නිෂ්පාදන (පාම් තෙල්/එළවළු තෙල්)	1	2	3	4	5	6
j) එළවළු (පලා ඇතුළු)	1	2	3	4	5	6
k) පළතුරු	1	2	3	4	5	6
l) සීනි/හකුරු	1	2	3	4	5	6
m) කුළුබඩු	1	2	3	4	5	6

n) රසකැවිලි (ටොෆි, චොක්ලට්, ආදිය)	1	2	3	4	5	6
o) පැණි බීම වර්ග / පළතුරු යුෂ	1	2	3	4	5	6
p) ක්ෂණික ආහාර	1	2	3	4	5	6

24. ඔබේ දරුවා පසුගිය සතියේදී පාසලේදී පහත සඳහන් දේ මිලදී ගත්තේද?

	**සෑම දිනම**	**3-4 වතාවක්**	**1-2 වතාවක්**	**කවදාවත් නැත**	**නොදනී**
a) පැණිබීම	1	2	3	4	5
b) අධික ලුණු සහිත අතිරේක ආහාර (උදා. රටකජු, බිස්කට්)	1	2	3	4	5
c) පැණිරස	1	2	3	4	5
d) වෙනත්: විශේෂයෙන් සඳහන් කරන්න	1	2	3	4	5

දරුවාගේ මාතෘ සමාජ ප්‍රාග්ධනය

25. ප්‍රජා කණ්ඩායම් සහ සාමාජිකත්වය:

1) පසුගිය මාස 12 ඇතුළත දරුවාගේ මව පහත සංගම් වල සාමාජිකත්වය දරා තිබේද?

2) පසුගිය මාස 12 තුළ, දරුවාගේ මව කිසියම් මානසික ආධාරයක්, ආර්ථික ආධාරයක් හෝ උපකාරයක් ලබා තිබේද?

**කණ්ඩායම් වර්ගය**	**1) ක්‍රියාකාරී සාමාජිකයෙකි**		**2) සහාය / උදවු**	
	**ඔව්**	**නැත**	**ඔව්**	**නැත**
1) වැඩ සම්බන්ධ / වෘත්තීය සමිති	1	2	1	2
2) ප්‍රජා සංවිධාන / සහයෝගීතාව	1	2	1	2
3) කාන්තා කණ්ඩායම්	1	2	1	2
4) දේශපාලන සංවිධාන	1	2	1	2
5) ආගමික කණ්ඩායම්	1	2	1	2
6) ණය / අවමංගල්‍ය කණ්ඩායම්	1	2	1	2
7) ක්‍රීඩා / සමාජ කණ්ඩායම්	1	2	1	2
8) තවත් අය: (විශේෂයෙන් සඳහන් කරන්න) ______	1	2	1	2

26. පසුගිය මාස 12 තුළ, දරුවාගේ මව කිසියම් මානසික ආධාරයක්, ආර්ථික ආධාරයක් හෝ උපකාරයක් ලබා තිබේද?

	ඔව්	නැත
1) පවුල	1	2
2) අසල්වැසියන්	1	2
3) අසල්වාසීන් නොවන මිතුරන්	1	2
4) ප්‍රජා නායකයින්	1	2
5) ආගමික නායකයා	1	2
6) දේශපාලනඥයින්	1	2
7) ප්‍රජා නිලධාරීන් / සිවිල් සේවයක්	1	2
8) පුණ්‍යායතන / රාජ්‍ය නොවන සංවිධාන	1	2
9) වෙනත්: විශේෂයෙන් සඳහන් කරන්න ________________________	1	2

27. පුරවැසි කටයුතු සහ සමාජ ප්‍රාග්ධන (මව)

	ඔව්	නැත
1) පසුගිය මාස 12 තුළදී ගැටලුවක් හෝ පොදු ප්‍රශ්නයක් විසඳීමට සමාජයේ අනෙකුත් සාමාජිකයන් සමඟ වැඩ කර තිබේද?	1	2
2) පසුගිය මාස 12 තුළ ප්‍රජාවේ ගැටලු පිළිබඳව පාලන අධිකාරියක් හෝ රාජ්‍ය සංවිධානයක් සමග කතා කළාද?	1	2
3) සාමාන්‍යයෙන් මේ ප්‍රජාවේ බහුතරයක් විශ්වාස කළ හැකිද?	1	2
4) මෙම ප්‍රජාවේ බහුතරයක් සාමාන්‍යයෙන් එකිනෙකා සමග කටයුතු කරන්නේද?	1	2
5) ඔබ ඇත්තටම මේ ප්‍රජාවේ කොටසක් ලෙස ඔබට දැනෙනවාද?	1	2
6) මෙම ප්‍රජාවෙහි බහුතරයක් ඔවුන්ට අවස්ථාවක් ලැබුණහොත් ඔබෙන් වාසියක් ලබා ගැනීමට උත්සාහ කරනු ඇතැයි ඔබ සිතනවාද?	1	2

ඔබගේ සහයෝගයට ස්තුතියි.
