# Supplementary material for: Double burden of maternal and child malnutrition and socioeconomic status in urban Sri Lanka
Source: PLoS One. 2019 Oct 22;14(10):e0224222. doi: 10.1371/journal.pone.0224222 (PMC6805006; doi:10.1371/journal.pone.0224222)
Supplement: S2 File — (PDF) [file pone.0224222.s002.pdf]

Serial no:						
School Name						
Type	1AB		1C		2	3
Grade		Class		Date		/ Sep /2017

Data collection sheet
A study on child malnutrition among primary school children in Gampaha district

**Anthropometric Measurements of the child**
1. Serial number : __________________<br>2. Height (cm) : _____________ cm<br>3. Weight (kg) : _____________ kg<br>4. Waist circumference (cm) : _____________ cm<br>5. MUAC (mm) : _____________ mm<br>6. Skinfold thickness (mm) :<br>6-1. Triceps : _____________ mm 6-2. Biceps : ____________ mm<br>6-3. Subscapular : ____________ mm 6-4. Suprailiac : _________________ mm

We are trying to find out about risk factors related to malnutrition among primary school children. Please answer following questions to the best of your knowledge.

<u>Remember:</u>

1. This is not a test. So there are no right and wrong answers.
2. Please answer all the questions as honestly and accurately as you can – this is very important.
3. Please use a pencil to fill in completely the column that goes with your answer choice.
4. Please use following instructions when answering,

   place a check mark (✔) in the square Eg.: ☑ Sinhalese
   put a circle (○) in the appropriate number Eg.: 1 ② 3 4
   write down your answer if there is no specific instruction.

5. Please refer to "Child Health Development Record" when filling the section of "Child Health Status" in this questionnaire,

Questionnaire

Child basic information	
1. Name (surname / first name)：	
2. Sex：1☐Girl 2☐Boy	3. Birthday (dd/mm/yy): ____/____/____<br>Age (dd/mm/yy): __/__/____ (on date)
4. Ethnic：1☐Sinhala 2☐Tamil 3☐Muslim 4☐Other	5. Language：1☐Sinhalese 2☐Tamil 3☐English 4☐Other
6. Religion：1☐Buddhist 2☐Hindu 3☐Islam 4☐Christian 5☐Other	

Child's Family structure
7. Who is the responder? 1☐Mother 2☐Father 3☐Guardian 4☐Other: specify(______________________)
8. Number of family members living together with the child*? 1TOTAL ________ (2Adult - ______ 3Child- ______ *except for the child)<br>Details：1☐Grandfather（__people） 2☐Grandmother（__people） 3☐Father 4☐Mother 5☐Older brother（__people）<br>6☐Older sister（__people） 7☐Younger brother（__people） 8☐Younger sister（__people） 9☐Other: specify(______________)
9. What is the average monthly income of your family?<br>1☐less than Rs.7,500 2☐Rs. 7,500 – < Rs,15,000 3☐Rs. 15,000 - < Rs.50,000 4☐more than Rs. 50,000 5☐Unknown
10. Please answer child's family structure (This family means parents and siblings of the child).

Family Structure	Example	a	b	c	d	e	f
**A) Relationship**<br>**1.Mother 2 Father**<br>**3.Older brother 4.Older sister**<br>**5.Younger brother**<br>**6.Younger sister 7.Other**	**1**<br>______	______	______	______	______	______	______
**B) Age（years old）**	40						
**C) Height and weight**	H 1 170 cm<br>W 2 70 kg	H 1____cm<br>W 2____kg	H 1____cm<br>W 2____kg	H 1____cm<br>W 2____kg	H 1____cm<br>W 2____kg	H 1____cm<br>W 2____kg	H 1____cm<br>W 2____kg
**D) Educational status**<br>*Choose one<br>**1.No education**<br>**2.Incomplete primary**<br>**3.Complete primary**<br>**4.Incomplete secondary**<br>**5.Complete secondary**<br>**6.Higher 7.Other**	1 2 ③<br>4 5 6<br>7	1 2 3<br>4 5 6<br>7	1 2 3<br>4 5 6<br>7	1 2 3<br>4 5 6<br>7	1 2 3<br>4 5 6<br>7	1 2 3<br>4 5 6<br>7	1 2 3<br>4 5 6<br>7
**E) Work status**<br>*Choose one of the main work<br>**1.Infant 2.Schooling**<br>**3.Job-hunting 4.Unemployment**<br>**5.Agriculture 6.Government**<br>**7.Private sector**<br>**8.Self-employment**<br>**9.Housekeeper 10.Other**	1 2 3<br>4 5 ⑥<br>7 8 9<br>10	1 2 3<br>4 5 6<br>7 8 9<br>10	1 2 3<br>4 5 6<br>7 8 9<br>10	1 2 3<br>4 5 6<br>7 8 9<br>10	1 2 3<br>4 5 6<br>7 8 9<br>10	1 2 3<br>4 5 6<br>7 8 9<br>10	1 2 3<br>4 5 6<br>7 8 9<br>10
**F) Health condition**<br>*Mark all that apply<br>**1.Diabetes 2.CKD**<br>**3.Dengue (in past 3 months)**<br>**4.Other (Specify)**	1 2 ③<br>4<br>______	1 2 3<br>4<br>______	1 2 3<br>4<br>______	1 2 3<br>4<br>______	1 2 3<br>4<br>______	1 2 3<br>4<br>______	1 2 3<br>4<br>______

Child Health Status

11. Do you have "Child Health Development Record"? 1☐Yes 2☐No

12. Birth weight： 1☐less than 2,500g 2☐2,500g - 4,000g 3☐> 4,000g 4☐Unknown

13. Immunization status（Mark all that apply）

After birth	0-4week	2 month	4 month	6 month	9 month	12 month	18month	3 years	5 years
Vaccine	1☐BCG	1☐OPV1<br>2☐DTwP1<br>3☐HepE1<br>4☐Hib1	1☐OPV2<br>2☐DTwP2<br>3☐HepE2<br>4☐Hib2<br>5☐IPV	1☐OPV3<br>2☐DTwP3<br>3☐HepE3<br>4☐Hib3	1☐LAJEV	1☐MMR1	1☐OPV4<br>2☐DTwP4	1☐MMR2	1☐OPV5<br>2☐DT1

14. Disease episode so far;（Mark all that apply）

1☐Pneumonia 2☐Asthma 3☐Malaria 4☐Influenza 5☐Anemia 6☐Worm infestation 7☐Diabetes 8☐Dengue

9☐Other: Specify (____________________)

15. Any health issues during last month? （Mark all that apply）

1☐Diarrhoea 2☐Vomitting 3☐Anaemia 4☐Headache 5☐Fever 6☐Weight loss 7☐Any infectious disease

8☐Traffic accident 9☐Other: specify (____________________)

16. What time normally does the child wake up and go to bed?: 1Go to bed（PM ____ : ____） 2Wake up（AM ____ : _____）

17. How many minutes does it take from your house to the school? (Choose one for each)

Type: 1☐on foot 2☐by bicycle 3☐by car 4☐by school bus 5☐by train 6☐Others: specify(________________________)

Minutes: 1☐less than 10minutes 2☐10～< 30minuites 3☐30～< 60minuites 4☐more than 60minuites

18. Does the child do physical activities except for the school physical class? (club activities, sport clubs etc.)

1☐No 2☐Yes → If yes, please specify the activity: ________________________________

19. Physical activity of the child: Please answer of free time physical activity frequencies and hours spent per one opportunity in a typical week.

Physical Activity	Frequency (per week)					Time spent for one opportunity				
	Almost everyday	5-6 times	3-4 times	1-2 times	Never	less than 30 mins	30-59 mins	1-2 hrs	2-3 hrs	More than 3 hrs
a) Vigorous-intensity activities (e.g. running and swimming)	1	2	3	4	5	a	b	c	d	e
b) Moderate-intensity activities (e.g. brisk walking and dancing)	1	2	3	4	5	a	b	c	d	e
c) Light-intensity activities (e.g. walking slowly)	1	2	3	4	5	a	b	c	d	e

20. Sedentary behaviour: How much time does the child usually spend sitting or reclining on a typical day?: __________ hours per day

Child's Behavior (Strengths and Difficulties Questionnaire)

21. For each item, please mark the box (Not True, Somewhat True or Certainly True.)It would help us if you answered all items as best you can even if you are not absolutely certain or the item seems daft! Please give your answers on the basis of the child's behavior over the last six months. (Choose one for each)	Not True	Somewhat true	Certainly true
A. Considerate of other people's feelings	1	2	3
B. Restless, overactive, cannot stay still for long	1	2	3
C. Often complains of headaches, stomach-aches or sickness	1	2	3
D. Shares readily with other children (treats, toys, pencils etc.)	1	2	3
E. Often has temper tantrums or hot tempers	1	2	3
F. Rather solitary, tends to play alone	1	2	3
G. Generally obedient, usually does what adults request	1	2	3
H. Many worries, often seems worried	1	2	3

I. Helpful if someone is hurt, upset or feeling ill	1	2	3
J. Constantly fidgeting or squirming	1	2	3
K. Has at least one good friend	1	2	3
L. Often fights with other children or bullies them	1	2	3
M. Often unhappy, down-hearted or tearful	1	2	3
N. Generally liked by other children	1	2	3
O. Easily distracted, concentration wanders	1	2	3
P. Nervous or clingy in new situations, easily loses confidence	1	2	3
Q. Kind to younger children	1	2	3
R. Often lies or cheats	1	2	3
S. Picked on or bullied by other children	1	2	3
T. Often volunteers to help others (parents, teachers, other children)	1	2	3
U. Thinks things out before acting	1	2	3
V. Steals from home, school or elsewhere	1	2	3
W. Gets on better with adults than with other children	1	2	3
X. Many fears, easily scared	1	2	3
Y. Sees tasks through to the end, good attention span	1	2	3

Child's Dietary Intake

22. Does your child usually take breakfast? : 1☐Yes 2☐No

23. How often did your child eat the following food items during the last week?

	Everyday	5-6 times	3-4 times	1-2 times	Never	Unknown
a) Rice and other cereals (finger millet)	1	2	3	4	5	6
b) Tubers (potato, sweet potato, cassava, etc.)	1	2	3	4	5	6
c) Bread (Chapati/ Roti)	1	2	3	4	5	6
d) Pulses / Dhal	1	2	3	4	5	6
e) Fish	1	2	3	4	5	6
f) Meat (beef, pork, chicken)	1	2	3	4	5	6
g) Eggs	1	2	3	4	5	6
h) Diary (curd, liquid milk, powder milk, etc.)	1	2	3	4	5	6
i) Coconut products , palm oil, vegetable oil, fat & etc.	1	2	3	4	5	6
j) Vegetables (including leaves)	1	2	3	4	5	6
k) Fruits	1	2	3	4	5	6
l) Sugar / Jaggary	1	2	3	4	5	6
m) Spices	1	2	3	4	5	6
n) Sweets (toffee, candy, chocolates, etc.)	1	2	3	4	5	6
o) Sweetened beverages / Juice / fizzy drinks	1	2	3	4	5	6
p) Fast food	1	2	3	4	5	6

24. Did your child buy any of the following at school during the last week?

	**Everyday**	**3-4 times**	**1-2 times**	**Never**	**Unknown**
**a) Soda**	1	2	3	4	5
**b) food with added salt (e.g. : peanut, biscuits)**	1	2	3	4	5
**c) Sweets**	1	2	3	4	5
**d) Other: Specify ____________________**	1	2	3	4	5

Maternal Social Capital of the child

25. Membership of community groups and support:

1) In the last 12 months, has child's mother been an active member of any of the following types of groups in your community?

2) In the last 12 months, has child's mother receive any emotional help, economic help or assistance from the group?

Group type	1) Active member		2) Support / Help	
	Yes	No	Yes	No
1) Work related/trade union	1	2	1	2
2) Community association/cooperation	1	2	1	2
3) Women's group	1	2	1	2
4) Political group	1	2	1	2
5) Religious group	1	2	1	2
6) Credit / funeral group	1	2	1	2
7) Sports/ social group	1	2	1	2
8) Others: specify ___________________________	1	2	1	2

26. In the last 12 months, has child's mother received any help or support from any of the following, this can be emotional help, economic help or assistance in helping you know or do things?

	Yes	No
1) Family	1	2
2) Neighbors	1	2
3) Friends who are not neighbors	1	2
4) Community leaders	1	2
5) Religious leader	1	2
6) Politicians	1	2
7) Government officials/civil service	1	2
8) Charitable organizations / NGO	1	2
9) Other: specify ________________________	1	2

27. Citizenship activities and cognitive social capital

	Yes	No
1) In the last 12 months, have child's mother joined together with other community members to address a problem or common issue?	1	2
2) In the last 12 months, have child's mother talked with a local authority or governmental organization about problems in this community?	1	2
3) In general, can the majority of people in this community be trusted?	1	2
4) Do the majority of people in this community generally get along with each other?	1	2
5) Do you feel as though you are really a part of this community?	1	2
6) Do you think that the majority of people in this community would try to take advantage of you if they got the chance?	1	2

Thank you very much for your cooperation.
